# Neuroprotective role of lactate in a human in vitro model of the ischemic penumbra

**DOI:** 10.1038/s41598-024-58669-5

**Published:** 2024-04-04

**Authors:** Marta Cerina, Marloes Levers, Jason M. Keller, Monica Frega

**Affiliations:** 1 Department of Clinical Neurophysiology, University of Twente, 7522 NB Enschede, The Netherlands; 2 Department of Biotechnology and Biosciences, University of Milano-Bicocca, piazza della Scienza 2, 20126 Milan, Italy; 3 Department of Human Genetics, Radboudumc, Donders Institute for Brain, Cognition, and Behaviour, 6500 HB Nijmegen, The Netherlands

**Keywords:** Cell biology, Neuroscience, Stem cells

## Abstract

In patients suffering from cerebral ischemic stroke, there is an urgent need for treatments to protect stressed yet viable brain cells. Recently, treatment strategies that induce neuronal activity have been shown to be neuroprotective. Here, we hypothesized that neuronal activation might maintain or trigger the astrocyte-to-neuron lactate shuttle (ANLS), whereby lactate is released from astrocytes to support the energy requirements of ATP-starved hypoxic neurons, and this leads to the observed neuroprotection. We tested this by using a human cell based in vitro model of the ischemic penumbra and investigating whether lactate might be neuroprotective in this setting. We found that lactate transporters are involved in the neuroprotective effect mediated by neuronal activation. Furthermore, we showed that lactate exogenously administered before hypoxia correlated with neuroprotection in our cellular model. In addition, stimulation of astrocyte with consequent endogenous production of lactate resulted in neuroprotection. To conclude, here we presented evidence that lactate transport into neurons contributes to neuroprotection during hypoxia providing a potential basis for therapeutic approaches in ischemic stroke.

## Introduction

Ischemic stroke, pathological condition caused by the sudden interruption of blood flow to the brain, is ranked as the second leading cause of death and a major cause of adult disability worldwide^1^. In the core of the infarct region, oxygen and glucose deprivation leads to irreversible neuronal dysfunction and tissue damage within minutes^2^. The area surrounding the core, the ischemic penumbra, is functionally silent but structurally intact and viable due to remaining blood flow from collateral vessels and thus has the potential to recover without damage, if normal blood perfusion is restored in time^3,4^. Otherwise, the tissue becomes progressively and irreversibly damaged, resulting in the expansion of the infarct region^3,4^. For this reason, the penumbra represents the most therapeutically relevant target in ischemic stroke research.

The only treatment proven to benefit patients is the early removal of the arterial occlusion (i.e. acute recanalization)^5^. In recent years, important advancements in stroke patients care allowed to gradually extend the therapeutic window for intervention (up to 24 h after stroke onset), and improved post-operation recovery and neurological outcomes^6,7^. However, not all patients are eligible for recanalization treatments, and the probability of enduring functional impairments is still about 50%, even if complete recanalization is achieved^6,7^*.* Thus, alternative, or adjunct neuroprotective strategies counteracting the molecular and cellular events causing ischemic damage have been sought^8^. The aim of neuroprotection is to slow down the pathophysiological cascade triggered by hypoxia in the ischemic penumbra, in order to promote neuronal survival, and support functional recovery and neurological improvement^9,10^. Over the past two decades, animal studies have shown that strategies directed at suppressing neuronal activity to preserve energy for basic cellular functions protect brain tissue^11^. However, in clinical trials promising pre-clinical studies have not been translated into positive outcomes^12,13^. Failure of translation to patients may be associated with the mechanism of action of treatments under study. In fact, contrary to the prevailing view on suppression, inhibiting neuronal activity during hypoxia has been associated with irreversible tissue damage both in vitro and in patients^14–17^. Alternatively, we have shown that neuronal activation had a neuroprotective effect (i.e. maintenance of electrophysiological activity and cellular viability) in a human in vitro model of the ischemic penumbra composed by neurons derived from human induced pluripotent stem cells (hiPSCs) exposed to oxygen – and consequent glucose—deprivation^18^. In the absence of neuronal activation, we observed a progressive decline of electrophysiological activity, functional connectivity and cell viability^3^ and—in line with the therapeutic window in patients—an irreversible neuronal network failure if re-oxygenation was not established within the first 24 h of hypoxia^6,7^.

The biological mechanisms underlying the benefit from neuronal activation in our human in vitro model of the ischemic penumbra are unknown.

Although lactate has long been considered a by-product of anaerobic metabolism, recent evidence showed that it can be found within the extracellular space of human neuronal tissue under aerobic conditions^19^, suggesting that it could have a physiological role in the brain as a supplemental energy source or even as a signalling molecule^20^. In this context, Pellerin and Magistretti proposed an astrocyte-to-neuron lactate shuttle (ANLS) hypothesis where astrocytes that respond to neuronal activity (e.g. via glutamate signalling) release lactate into the extracellular space to energetically support neurons^21,22^. Recent evidence also suggests that lactate might be a neuroprotective agent in certain pathological contexts. Studies in animal models of ischemic stroke showed that lactate, either endogenously produced during hypoxia or applied exogenously at reoxygenation, supports energy metabolism and functional recovery in neurons^23–26^, and that direct intracerebroventricular or intravenous injection of lactate led to a decrease in infarct volume and an improvement in neurological outcome^26,27^. Moreover, it has been demonstrated that lactate mediates neuroprotection through different mechanisms of action which are relevant in ischemic stroke (i.e. support to energy metabolism^23–25^, counteraction of glutamate excitotoxicity^28,29^, oxidative stress^30–32^ and cellular death^33,34^, expression of plasticity-related and pro-survival genes ^33,34^, and protection of astrocytes^35^). According to the ANLS hypothesis, lactate production and release by astrocytes is dependent on neuronal activity^21,22^. Therefore, we hypothesised that neuronal activation might maintain or trigger the ANLS to protect hypoxic neurons. In support of our hypothesis, a recent work from Batthi and Frostig showed the critical role of neuronal lactate transporters in neuroprotection mediated by sensory stimulation of the ischemic area in a rat model of ischemic stroke^36^. Here, we investigated the mechanisms underlying the neuroprotective effect of neuronal stimulation during hypoxia observed in the human in vitro model of the ischemic penumbra previously published by us^18^. We found that lactate transporters are critically involved in the neuroprotective effect mediated by neuronal activation during hypoxia. Furthermore, we showed that lactate exogenously administered before hypoxia correlates with neuroprotection in our model. Finally, we observed that activation of astrocytes with consequent endogenous production of lactate is associated with neuroprotection.

## Results

### Neuronal activation protects cells during hypoxia

**Figure 1:**
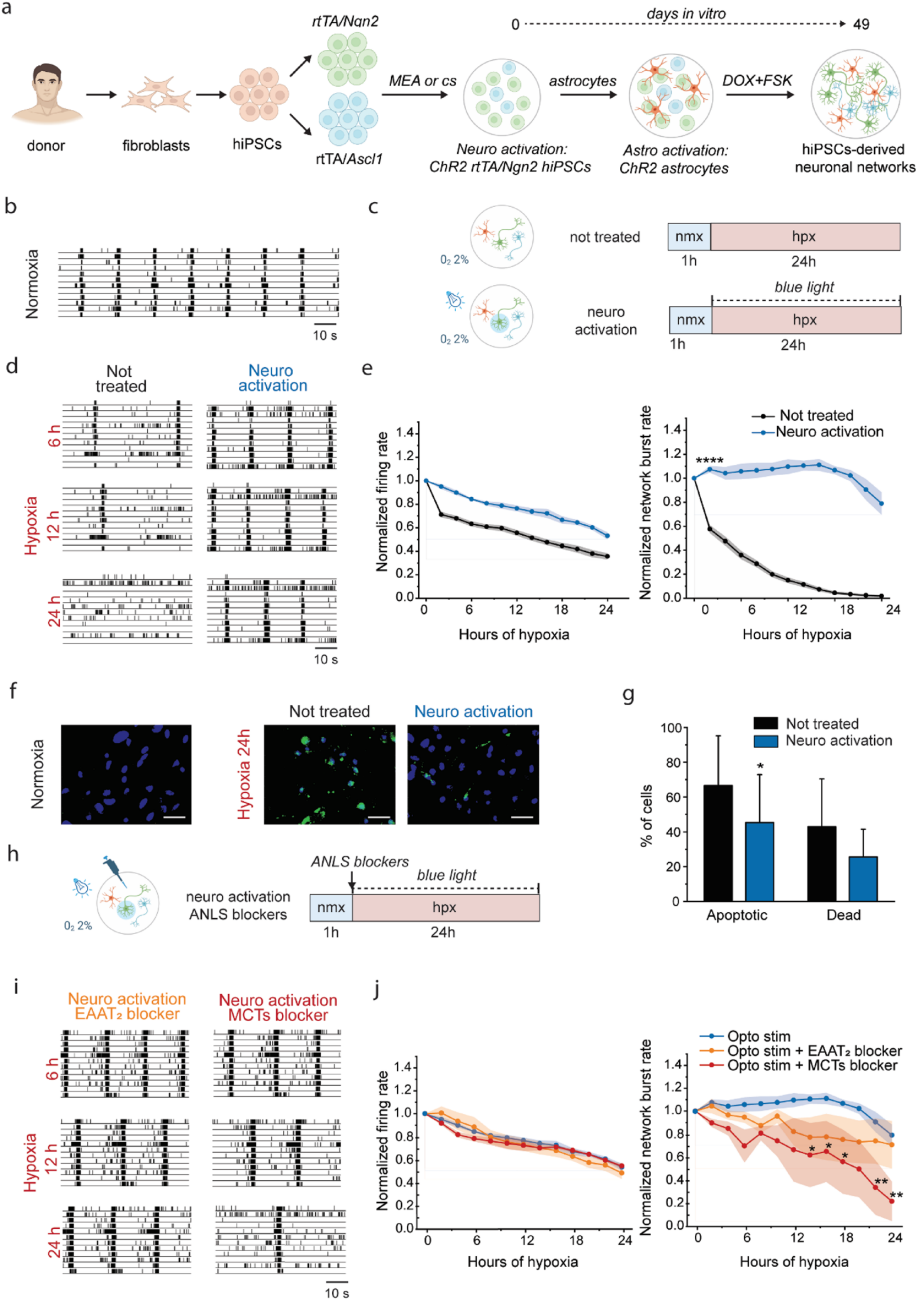
Lactate transport is involved in neuroprotection mediated by neuronal activation. (**a**) Schematic presentation of the differentiation protocol of hiPSCs reprogrammed from fibroblasts of healthy donors. (**b**) Representative raster plots showing 3 min of electrophysiological activity exhibited in normoxia by functionally mature healthy neuronal networks. (**c**) Schemes showing the experimental protocol, indicating the duration of normoxia (nmx, blue) and hypoxia (hpx, red), and experimental conditions during hypoxia (i.e. not treated and activated with optogenetic stimulation (neuro activation)). (**d**) Representative raster plots showing 60 s of electrophysiological activity exhibited at different timepoints of hypoxia (6, 12, 24 h) by neuronal networks not treated and activated with optogenetic stimulation (neuro activation). (**e**) Graphs showing the effect of 24 h of hypoxia on MFR and NBR in neuronal networks not treated and activated with optogenetic stimulation. The values are normalized to the data of normoxia (not treated n = 20, neuro activation n = 5). ****p < 0.0001, two-way ANOVA test and post hoc Bonferroni correction wa performed between conditions. Asterisks are not shown after the first timepoint the corresponding p-value was found. (**f**) Representative images of live/dead assay in normoxia and after 24 h of hypoxia in neuronal networks not treated or activated with optogenetic stimulation. Neurons were stained for DAPI (blue, nuclei of all cells) and CellEvent™ Caspase-3/7 Green Detection Reagent (green, apoptotic cells). Scale bar: 20 μm. (**g**) Bar graphs showing the percentage of apoptotic cells and dead cells in hiPSCs-derived neurons not treated and activated with optogenetic stimulation (not treated n = 16 pictures per 2 cultures, neuro activation n = 16 pictures per 2 cultures). *p < 0.05, Mann Whitney test was performed between conditions. (**h**) Scheme showing the experimental protocol, indicating the duration of normoxia (nmx, blue) and hypoxia (hpx, red), and experimental condition during hypoxia (i.e. activated with optogenetic stimulation with, alternatively, EAAT2 blocker or MCTs blocker (neuro activation + ANLS blockers)). (**i**) Representative raster plots showing 60 s of electrophysiological activity exhibited at different timepoints of hypoxia (6, 12, 24 h) by neuronal networks activated with optogenetic stimulation with, alternatively, EAAT2 blocker or MCTs blocker. (**j**) Graphs showing the effect of ANLS blockers on MFR and NBR in neuronal networks activated with optogenetic stimulation. The values are normalized to the data of normoxia (neuro activation n = 5, neuro activation + EAAT2 blocker n = 5, neuro activation + MCT1/2 blocker n = 3). *p < 0.05, **p < 0.005, two-way ANOVA test and post hoc Bonferroni correction was performed between conditions.

We differentiated hiPSCs into human neuronal networks (Fig. 1a) and investigated the effect of artificial activation during 24 h of low oxygen exposure on neuronal activity and viability. By seven weeks in vitro, hiPSCs-derived neuronal networks grown on Micro Electrode Arrays (MEAs) were functionally mature, showing typical neuronal morphology and stable electrophysiological activity composed of spikes and synchronous bursts^18,37,38^ (Fig. 1b). To ensure consistency between cell cultures, all hiPSCs-derived neuronal networks were used in hypoxia experiments at this stage of maturation^39^. When exposed to low oxygen, both the firing rate (MFR) and network bursting activity (NBR) of the networks progressively declined, leading to a complete inhibition of network bursts within 24 h (Fig. 1c–e). In line with our previous results^18^, we found that when networks were artificially activated during hypoxia the MFR was maintained at higher levels compared to untreated neuronal networks (Fig. 1c–e). Additionally, the NBR was preserved at baseline levels for the first 18 h of hypoxia (p > 0.999), and it was significantly higher at every timepoint compared to untreated neuronal networks (p < 0.0001 at each timepoint, Fig. 1d,e). Next, we evaluated the effect of neuronal activation on cell viability by determining the percentage of apoptotic and dead cells after 24 h of hypoxia (Fig. 1f). Corroborating our MEA recordings, we found a significantly higher percentage of apoptotic cells in untreated neuronal networks compared to those where neuronal activation was applied during hypoxia (p < 0.05, Fig. 1g). However, the percentage of dead cells was not statistically different between the two experimental groups (p > 0.9999, Fig. 1g).

### Lactate transporters are involved in neuroprotection mediated by neuronal activation during hypoxia

We turned our attention to the biological mechanisms underlying the neuroprotective effect that artificial activation has during hypoxia. To evaluate this, we pharmacologically blocked transporters that are predicted to be directly involved in the ANLS (Fig. 1h). In particular, we blocked excitatory amino acid transporter 2 (i.e. EAAT2 or GLT-1, responsible for glutamate uptake into astrocytes^22,40^), and two monocarboxylate transporters (i.e. MCT1 and MCT2, the major lactate transporters present on neurons as well as glial cells^41^). The effects of the blockers were primarily observable on the NBR: inhibiting EAAT2 slightly impaired neuroprotection at the highest concentration tested (Fig. 1i,j, Suppl. Fig. 1), whereas blocking MCT1/2 showed a strong inhibition of neuroprotection (p < 0.05 after 14, 16 and 18 h, p < 0.005 after 22 and 24 h, Fig. 1i,j).

### Exogenous lactate administrated before hypoxia is neuroprotective

Since blocking MCT1/2 impaired the neuroprotective effect mediated by neuronal activation, we postulated that lactate itself, whose production and release by astrocytes could be maintained or triggered by neuronal activation, may have a neuroprotective effect on its own in our model.

**Figure 2:**
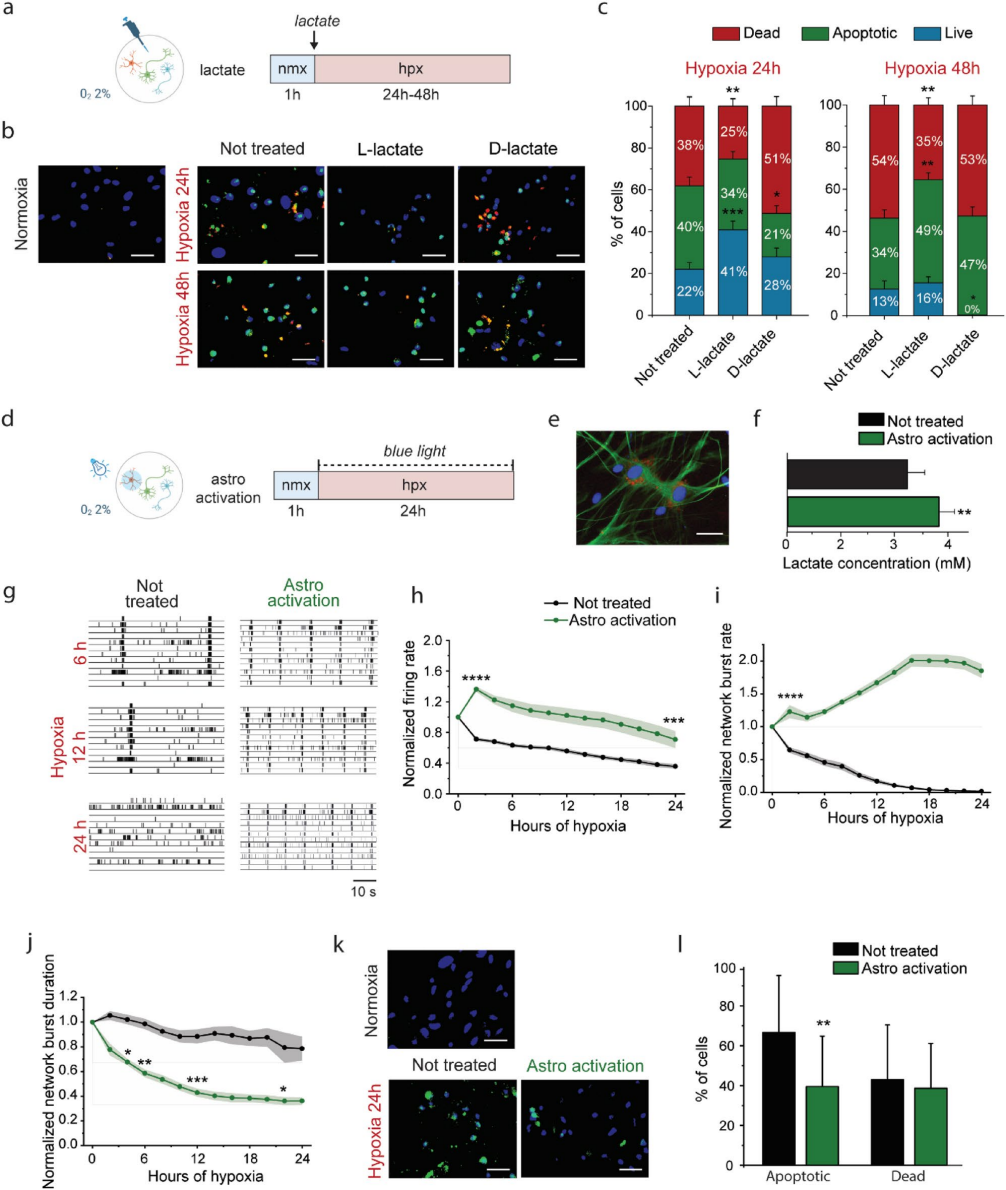
Lactate is neuroprotective in neuronal networks exposed to hypoxia. (**a**) Scheme showing the experimental protocol, indicating the duration of normoxia (nmx, blue) and hypoxia (hpx, red), and experimental condition during hypoxia (i.e. treated with lactate before hypoxia). (**b**) Representative images of live/dead assay in normoxia and after 24 and 48 h of hypoxia in hiPSCs-derived neurons not treated and treated with 10 mM of l-lactate and 10 mM of d-lactate stained for DAPI (blue), CellEvent™ Caspase-3/7 Green Detection Reagent (green) and PI (red). Scale bar: 20 μm. (**c**) Stacked column graphs showing the percentage of live, apoptotic and dead cells (not treated n = 56 pictures per 7 cultures, l-lactate 10 mM n = 40 pictures per 5 cultures, D-lactate 10 mM n = 16 pictures per 2 cultures). *p < 0.05, **p < 0.005, ***p < 0.0005, unpaired t test or Mann Whitney test was performed between conditions. (**d**) Scheme showing the experimental protocol, indicating the duration of normoxia (nmx, blue) and hypoxia (hpx, red), and experimental condition during hypoxia (i.e. activation of astrocytes (astro activation)). (**e**) A representative image showing hiPSCs-derived neurons with rodent astrocytes stained for GFAP (green), cell nuclei stained with DAPI (blue), ChR2 (red). (**f**) Bar graph showing lactate concentration in the medium collected from astrocytic cultures not treated and activated with optogenetic stimulation. (**g**) Representative raster plots showing 60 s of electrophysiological activity exhibited at different timepoints of hypoxia (6, 12, 24 h) by neuronal networks not treated and with activation of astrocytes. (**h-j**) Graphs showing the effect of 24 h of hypoxia on MFR, NBR and NBD in neuronal networks not treated and with activation of astrocytes. The values are normalized to the data of normoxia (not treated n = 20, astro activation n = 9). *p < 0.05, **p < 0.005, ***p < 0.0005, ****p < 0.0001, two-way ANOVA test and post hoc Bonferroni correction was performed between conditions. Asterisks are not shown after the first timepoint the corresponding p-value was found. (**k**) Representative images of live/dead assay in normoxia and after 24 h of hypoxia in neuronal networks not treated or with activation of astrocytes. Neurons were stained for DAPI (blue, nuclei of all cells) and CellEvent™ Caspase-3/7 Green Detection Reagent (green, apoptotic cells). Scale bar: 20 μm. (**l**) Bar graphs showing the percentage of apoptotic and dead cells in hiPSCs-derived neurons not treated and with activation of astrocytes (not treated n = 16 pictures per 2 cultures, astro activation n = 16 pictures per 2 cultures). **p < 0.005, Mann Whitney test was performed between conditions.

First, we tested this hypothesis by adding l-lactate (5 mM, 10 mM and 20 mM) to the culture medium of neuronal networks before exposing them to low oxygen (Fig. 2a). We found that L-lactate immediately blunted the electrophysiological activity of hiPSCs-derived neuronal networks before hypoxia onset, in a concentration-dependent manner (Suppl. Fig. 2a). Therefore, we decided to investigate the potential neuroprotective effect of l-lactate by evaluating the percentage of live, apoptotic and dead cells after 24 and 48 h of hypoxia.

After 24 h of hypoxia, neuronal networks treated with 10 mM of l-lactate showed a higher percentage of live cells, plus a lower percentage of apoptotic and dead cells, as compared to neuronal networks exposed to hypoxia without lactate supplementation (Fig. 2b,c). Differences in the numbers of live and dead cells were statistically significant (p < 0.0005 and p < 0.005, respectively), whereas the quantity of apoptotic cells was not (p > 0.9999). After 48 h of hypoxia, the percentage of live cells remaining in neuronal networks treated with l-lactate was very similar to untreated neuronal networks (p > 0.9999). However, the percentage of apoptotic cells was significantly higher (p < 0.005), and the percentage of dead cells was significantly lower (p < 0.005, Fig. 2b,c). These results suggest that adding 10 mM of l-lactate to the culture medium before hypoxia delayed apoptosis and consequent cell death. However, we found that lower or higher concentrations of l-lactate (i.e. 5 mM and 20 mM) had no measurable benefit on cell viability in our test system. While 5 mM of l-lactate did not have any deleterious or positive effects on the percentage of live cells, 20 mM of l-lactate appeared to be neurotoxic by increasing the number of apoptotic and dead cells (Suppl. Fig. 2b).

Next, to discriminate whether the potential neuroprotective effect of lactate depends on its modulatory effect of electrophysiological activity, on its use as a metabolic substrate, or on other mechanisms, we compared the effects of the addition of d-lactate (10 mM) and l-lactate. The D enantiomer of lactate, indeed, is poorly metabolized by neurons^42^, but it can be up taken by neurons via MCT1^41^ and is a partial agonist of the HCA1 receptor (i.e. responsible for the modulation of neuronal activity^43^). As was observed for l-lactate, d-lactate also immediately inhibited electrophysiological activity (Suppl. Fig. 2a). At 24 h of hypoxia, we found a higher percentage of dead cells in neuronal networks treated with D-lactate compared to untreated neuronal networks (Fig. 2b,c). After 48 h of hypoxia, the percentages of both apoptotic and dead cells in cultures treated with D-lactate were higher than in untreated neuronal networks, and the percentage of live cells was close to zero (Fig. 2b,c).

### Lactate is released by astrocyte activation and correlates with neuroprotection

We observed that the administration of lactate before hypoxia onset correlated with neuroprotection. Then, we decided to investigate whether lactate released by astrocytes during hypoxia had a similar effect.

To induce lactate release from astrocytes, we transduced the cells with Channelrhodopsin-2 (ChR2) and stimulated them optogenetically with blue light^44^ (Fig. 2d). ChR2 integration was cell-specific and successful, as shown by ChR2 expression only in astrocytes (Fig. 2e). We found that the concentration of lactate was significantly higher in the medium of cultures underwent astrocyte activation (p < 0.005, Fig. 2f), confirming that stimulation induced lactate release from astrocytes.

When exposed to 2% oxygen, we observed higher MFRs and NBRs in neuronal networks where astrocytes were activated optogenetically as compared to untreated cultures (p < 0.0001 during the first 22 h of hypoxia and p < 0.005 afterwards for MFR, p < 0.0001 at each timepoint for NBR, Fig. 2g–i). Moreover, astrocyte activation shortened the duration of network bursts (NBD), as compared to not treated neuronal networks, at each timepoint during the hypoxic period (Fig. 2g,j).

Next, we investigated the effect of astrocyte activation on cell viability, by evaluating the percentage of apoptotic and dead cells after 24 h of hypoxia. We found that the percentage of apoptotic cells in neuronal networks where astrocytes were activated was significantly lower compared to untreated networks (p < 0.05, Fig. 2k,l). However, the percentage of dead cells was not statistically different between the two test conditions (p > 0.9999, Fig. 2k,l).

## Discussion

In this study, we used hiPSCs-derived neuronal networks exposed to hypoxia as a human in vitro model of the ischemic penumbra to investigate whether the ANLS is involved in the neuroprotective effect mediated by neuronal activation. We found that the blockade of lactate transporters impairs the neuroprotective effect mediated by neuronal activation, clearly indicating that lactate transport is involved in neuroprotection. Furthermore, we observed that supplementing l-lactate, but not d-lactate, in the culture medium prior to the onset of hypoxia had a neuroprotective effect with respect to cell viability. This implies that lactate is neuroprotective in our model of the ischemic penumbra, and that this beneficial effect is mediated by the transport and metabolism of lactate in neurons. Lastly, we showed that optogenetic stimulation of astrocytes during hypoxia induced released of lactate and was correlated with neuroprotection, as demonstrated by the preservation of both spontaneous electrophysiological activity and cell viability.

We showed that the blockade of two transporters involved in the ANLS partially impairs the neuroprotective effect mediated by neuronal activation via optogenetic stimulation. We found that inhibiting EAAT2, which mediates glutamate uptake into astrocytes, a trigger for lactate production and release into the extracellular space^22,40^, only mildly affected the optogenetic stimulation effect on synchronized activity in a concentration dependent manner. On the contrary, blocking MCT1/2, which mediate lactate transport at both neuronal and astrocytic level, showed a strong inhibition of neuroprotection. These results clearly showed that lactate transport via MCTs plays a critical role in neuroprotection mediated by neuronal activation in hypoxia, indicating that neuronal activation induced either lactate release by astrocytes and/or lactate uptake by neurons. Our results are in line with those of Schurr et al. who demonstrated that the blockade of lactate transport exacerbates neuronal damage after ischemia in a rodent model of global ischemia^45^. Furthermore, our results are supported by a recent work published by Bhatti and Frostig, who showed the pivotal role of neuronal lactate transporters (i.e. MCT2) in neuroprotection mediated by sensory stimulation in a in vivo model of ischemic stroke. In contrast to our in vitro human model of the ischemic penumbra consisting in the combination of hiPSCs-derived neurons and rat astrocytes exposed to hypoxia and consequent low level of glucose, Bhatti and Frostig used an in vivo rat model in which the entire system of neurons, astrocytes, and blood flow is modified after permanent middle cerebral artery occlusion (pMCAo), which results in both hypoxic conditions and minimal glucose supply through major changes in collateral blood flow^36^. In both models, the neuroprotective effect mediated by the neuronal stimulation (i.e. optogenetic stimulation in vitro and whisking stimulation in vivo) was hampered when lactate transporters were blocked. In particular, an increase of the volume of the necrotic infarct core was found in vivo^36^, while a loss of neuronal functionality and cellular viability was observed in vitro. These are two ways to evaluate neuroprotection: it is the progressive transformation of the penumbra into irreversible damaged tissue, which goes through the impairment of neuronal function and the activation of apoptosis towards cell death, which accounts for the expansion of the infarct core’s volume^3^. The fact that the critical role of MCTs in neuroprotection for stroke has been shown in two different models increases the trustworthiness of the results and might advance stroke patient care.

Results obtained with the blockade of MCTs suggested that lactate in itself might be neuroprotective during hypoxia. Therefore, we decided to test this hypothesis by supplying neurons with lactate in two different ways: (i) by administering lactate before hypoxia onset, and (ii) by inducing lactate release through optogenetic stimulation of astrocytes. In both ways, we observed a neuroprotective effect in our model. In line with previous results obtained in rodent neuronal cultures^43^, we found that the administration of lactate in a concentration range of 5–20 mM (which are relatively high as compared to physiological ones^19^) caused an immediate drop in the electrophysiological activity of hiPSCs-derived neuronal networks, in a concentration-dependent manner. To our knowledge, this is the first study demonstrating that lactate has a modulatory effect on activity of human neurons. Both l-lactate and d-lactate had the same effect on electrophysiological activity, since they modulate neuronal activity by binding the same receptor on the plasma membrane^43^. For this reason, we decided to assess the potential neuroprotective effect of l-lactate and d-lactate on cell viability. Our results showed that treatment with 10 mM of l-lactate induces a delay in apoptosis, and subsequent death, of neuronal cells exposed to hypoxia. Conversely, in neuronal networks treated with d-lactate we observed an increase in apoptosis and cell death as compared to not treated cultures. Since d-lactate can modulate neuronal activity but cannot be metabolized by neuronal cells, its failure as a neuroprotective agent allowed us to draw two conclusions. Firstly, the neuroprotective effect of l-lactate is not, or at least not only, dependent on the partial silencing of electrophysiological activity but depends on its use as a metabolic substrate. Secondly, d-lactate neurotoxic effect indicates that suppression of neuronal activity during hypoxia alone is not effective in protecting human neurons from ischemic damage, which is consistent with the above-mentioned failure of neuroprotective strategies directed to suppression of neuronal activity during hypoxia^12,13^. Moreover, because the MCTs carries both l-and d-lactate, the latter might act as a competitive inhibitor of l-lactate transport, and so reducing neuroprotection mediated by the uptake of l-lactate already present in the medium.

For a long time, lactate in the adult brain has not been considered more than a mere by-product of anaerobic metabolism. However, recent evidence suggested that lactate has a physiological role in the brain as a supplementary energy source and signaling molecule^20^. Several studies demonstrated that lactate not only is able to sustain neuronal activity in different models, but also that its use as energy substrate is preferred over glucose when neuronal activity is increased^46–48^. In particular, a recent work from Dembitskaya et al. demonstrated that glucose and lactate metabolism are differentially engaged in neuronal fueling depending on the complexity of the activity-dependent plasticity and behavior in in vivo rodent models^48^. They found that lactate is mandatory for demanding neural computation and cognitive task requiring high attentional load, while glucose is sufficient for lighter forms of activity-dependent long-term potentiation. These findings support our results showing that when neurons are activated, lactate metabolism is involved.

In addition, it has been shown that lactate has a neuroprotective effect in pathological conditions in which the availability of energy substrates is limited, such as cerebral ischemic stroke. Studies on in vitro rodent models, showed that lactate, either endogenously produced during hypoxia or applied exogenously at the end of it, can be metabolically used by neurons, and it is preferential to glucose for functional recovery during the reoxygenation period^23–26^. In in vivo models, Berthet et al. showed that either intracerebroventricular and intravenous injection of lactate after reperfusion led to a significant decrease in lesion size and an improvement in neurologic outcome in a rat model of cerebral ischemia^26,27^.

According to literature, lactate contributes to neuroprotection through different mechanisms of action. Firstly, lactate represents a convenient metabolic substrate for energy-deprived neurons, since, as compared to glucose, its use reduces the strain on the depleted energy levels. Secondly, lactate counteracts glutamate excitotoxicity^28,49,50^, by triggering well-coordinated mechanisms leading to hyperpolarization of neurons, and thus to a decrease of neuronal excitability^50^, and increased expression of anti-apoptotic factors^51^. Furthermore, recent studies have investigated the neuroprotective effect of lactate against oxidative stress, showing that lactate triggers antioxidant defenses and pro-survival pathways, through the production of NADH, reduction of intracellular pH and a mild Reactive Oxygen Species (ROS) burst^30–32^. Thirdly, it has been demonstrated that physiological concentrations of lactate only, not glucose or pyruvate, promote the expression of key plasticity-related genes^33^ and that lactate promotes the upregulation of pro-survival genes and the downregulation of pro-death genes in an NMDARs-dependent manner, revealing a transcriptome profile favoring neuroprotection^34^. Fourthly, lactate exerts its neuroprotective effect by targeting astrocytes. In particular, Benerjee et al. demonstrated that ischemic concentrations of lactate promote TWIK-related potassium channel (TREK1) expression, which contributes to maintain astrocytic properties (i.e. conductance, resting membrane potential, potassium buffering, glutamate uptake and pH), underlying ionic homeostasis and excitotoxicity prevention^35^.

Since the pathophysiological cascade of ischemic stroke is incredibly complex, a neuroprotective agent targeting more than one pathophysiological event could represent a successful approach to develop clinically relevant therapies for ischemic stroke. Actually, a few studies investigated treatment strategies with multiple therapeutic targets providing strong proof of principle of their effectiveness in neuroprotection in ischemic stroke^52–54^. In addition, we suggest that lactate represents a convenient neuroprotective agent in the perspective of translation to clinic since it can be administrated to patients in a simple way which is intravenous supplementation. In recent years, the administration of lactate enriched solutions has been studied in healthy subjects, and in acute heart failure and severe traumatic brain injured patients, showing beneficial effects, and no severe adverse event^55^.

Astrocyte activation during hypoxia, with the subsequent release of small concentrations of lactate in the extracellular space, was also associated to neuroprotection in our model. In particular, astrocyte stimulation resulted in the decrease of apoptotic and dead cells, as compared to not treated neuronal networks, and the preservation of electrophysiological activity throughout the 24 h of hypoxia. The release of small concentrations of lactate over time had a very different effect as compared to the complete abolishment of activity observed when high concentrations of lactate were exogenously administered at once. In fact, with astrocyte activation the activity was finely modulated during hypoxia: we observed an increase in the frequency of network bursts, and a shortening of their duration. Both the balance AMPARs/NMDARs-mediated currents, and excitation/inhibition has been found to regulate the network burst activity in hiPSCs-derived neuronal networks^37,38^. Since lactate potentiates NMDARs-mediated currents^33^ and has an inhibitory effect on neuronal activity^43^, a change in the frequency and duration of network bursts can be associated with the effect of lactate on neurons.

Even though we confirmed that astrocyte activation induces lactate release in the extracellular space, we cannot exclude that other mechanisms might be activated by optogenetic stimulation of astrocytes. In fact, astrocytes in the brain are involved in numerous critical functions in support of neuronal functionality and viability in both physiological and pathological contexts, including ischemic stroke^56–58^. During the acute phase of ischemic stroke, astrocytes undergo important morphological modifications and perform multiple functions both detrimental and beneficial for neuronal survival^59^. Within minutes after injury, activated astrocytes, also referred to as reactive astrogliosis, produce and release inflammatory mediators^60^ and ROS^61^, which may induce apoptosis and cell death. However, they also release neurotrophic factors in support of neurons^62^ and they contribute to neuroprotection against glutamate excitotoxicity^63^ and oxidative stress^64,65^. During the late recovery phase after stroke, astrocytes participate in neurogenesis^66^, synaptogenesis^67,68^, angiogenesis^69^, and thereby promote neurological recovery. For all these reasons, neuroprotective strategies that specifically target neurons alone may be insufficient to improve neurological outcome after ischemic stroke. In the past years, several strategies directed at reducing the detrimental effects and amplifying the beneficial effects of astrocytes on neuroprotection and neurorestoration have been investigated^70,71^. Some of those approaches aimed, for instance, to protect astrocyte viability, enhance their ability to protect neurons from excitotoxicity and oxidative damage, and promote the release of neurotrophins and other factors for neuro- and angiogenesis^70,71^. Developing treatment strategies that target multiple cell types, and in particular astrocytes, may represent a successful approach in ischemic stroke.

In this study, we investigated the biological mechanisms underlying the neuroprotective effect mediated by neuronal activation in a hiPSCs-based in vitro model of the ischemic penumbra. We found that (i) lactate transport is critically involved in the neuroprotective effect mediated by neuronal activation in hypoxia, (ii) lactate supplemented in the culture medium had a neuroprotective effect, and (iii) astrocyte stimulation with consequent release of lactate was associated with neuroprotection. Although non-invasive ways to stimulate neuronal activity in patients are available (e.g. transcranial magnetic stimulation or transcranial direct-current stimulation, noninvasive vagus nerve stimulation) and there is interest in using them for treatment of stroke patients^72^, they are expensive and not easy to technically perform. Since the involvement of lactate transporters in the neuroprotective effect mediated by stimulation has been observed not only in our in vitro model but also in an in vivo rat model^36^ and we have found that lactate itself has a neuroprotective effect in ischemic stroke, it might be a valid candidate approach for neuroprotection which exerts its effect on multiple therapeutic targets and can be easily administrated to patients.

## Methods

### hiPSCs generation and neuronal differentiation

We used two previously characterized hiPSCs lines^37^, kindly provided by Mossink et al. (Radboud University Medical Centre). Both lines were generated from fibroblasts. Control line 1 (C1, healthy 30 years old female) was reprogrammed via episomal reprogramming (Coriell Institute for medical research, GM25256). Control line 2 (C2, healthy 51 years old male) was reprogrammed via a non-integrating Sendai virus (KULSTEM iPSC core facility Leuven, Belgium, KSF-16-025). We received the hiPSCs lines in frozen vials. The research was conducted in accordance with the principles embodied in the Declaration of Helsinki and in accordance with local statutory requirements. All participants gave written informed consent to participate in the study (Coriell Institute for medical research, GM25256 and KULSTEM iPSC core facility Leuven, Belgium, KSF-16-025). hiPSCs were cultured on Matrigel (Corning, #356237) in E8 flex medium (Thermo Fisher Scientific) supplemented with puromycin (0.5 µg/ml, Sigma Aldrich) and G418 (50 µg/ml, Sigma Aldrich) at 37 °C/5% CO_2_. Medium was refreshed every 2–3 days and cells were passaged twice per week using an enzyme-free reagent (ReLeSR, Stem Cell Technologies).

HiPSCs were directly derived into Glutamatergic cortical layer 2/3 neurons and GABAergic neurons by overexpressing mouse neuronal determinant Neurogenin 2 (*Ngn2*) and Achaete-scute homolog 1 (*Ascl1*) upon doxycycline treatment^37,73^, respectively as described in Pires Monteiro et al.^18^. Rodent astrocytes were isolated from brain cortices of new-born (P1) Wistar rats (Janvier-Labs). All surgical and experimental procedures regarding animal primary cell lines followed Dutch and European laws and guidelines, and were approved by the Centrale Commissie Dierproeven (CCD) (AVD11000202115663). All methods are reported in accordance with the ARRIVE guidelines. Two days after plating (i.e. day in vitro*,* DIV 2), rodent astrocytes were added in a 1:1 ratio. On DIV 3, the medium was replaced with Neurobasal medium (Thermo Fisher Scientific) supplemented with B-27 (Thermo Fisher Scientific), glutaMAX (Thermo Fisher Scientific), primocin (0.1 μg/ml, Invivogen), doxycycline (4 µg/ml, Sigma Aldrich), BDNF (10 ng/ml, Bioconnect), NT3 (10 ng/ml, Bioconnect) and forskolin (10 μM, Sigma Aldrich). Cytosine β-d-arabinofuranoside (Ara-C) (2 µM, Sigma Aldrich) was added to remove any proliferating cells. From this day onwards, half of the medium was refreshed 3 times a week. The medium was additionally supplemented with 2.5% FCS (Sigma Aldrich) from DIV 9 onwards. Doxycycline and forskolin were removed after DIV 13. Neuronal networks were kept at 37 °C, 5% CO_2_ until the day of experiment.

### Experimental protocol

The experiments were conducted at 7 weeks in vitro in a computer-controlled incubator (temperature 37 °C, 40% humidity, 5% CO_2_). After 30 min of accommodation in normoxia (oxygen 20%), cultures were exposed to 24 or 48 h of hypoxia (oxygen 2%). Neuronal network activity was recorded with MEA for 10 min in normoxia and once every two hours in hypoxia. Cell viability was assessed in normoxia and after 24 and 48 h of hypoxia.

During hypoxia, three experimental conditions reported below were used.

1. *Neuronal and astrocyte activation.* It was performed through optogenetic stimulation of excitatory neurons and astrocytes. To make excitatory neurons or astrocytes sensitive to blue light, 1 h after plating (DIV 0 and DIV 2 for neurons and astrocytes, respectively) *Ngn2*-hiPSCs or astrocytes were transduced with an adeno-associated virus (AAV) serotype 2 encoding Channelrhodopsin-2 (ChR2) (AAV2-hSyn-hChR2(H134R)-mCherry, UNC Vector Core)^74^ and AAV serotype 2 encoding ChR2 (AAV-GFAP-hChR2(H134R)-mCherry, UNC Vector Core)^44,75^, respectively. Afterwards, culture medium was replaced. On the day of the experiment, blue light (λ = 470 nm) was delivered to neuronal networks during the entire hypoxic period with the use of a Multiwell-Optogenetic prototype (Multi Channel Systems, Reutlingen, Germany) connected with the computer through the interface board. Light pulses (0.2 Hz, 10 mA for neuronal activation; 25 Hz, 0.3 mA for astrocyte activation) were applied for 3 min every 2 h, as in Ref.^18^. Experiments in which blue light stimulation was delivered to neuronal networks not transduced with ChR2 were performed. Our results showed that these networks responded to hypoxia as the not treated ones (Suppl. Fig. 2c).
2. *Blockage of ANLS transporters.* Dihydrokainic acid (DHK) (Sigma-Aldrich) (i.e. EAAT2 selective inhibitor) was used at a final concentration of 300 μM^76^ and AR-C155858 (Sigma-Aldrich) (inhibitor of monocarboxylate transporters (MCTs) 1 and 2) was pre-diluted in DMSO and used at a final concentration of 1 μM^77^. The blockers were added in the medium independently before the onset of hypoxia.
3. *Addition of Lactate.* Sodium l-lactate and sodium d-lactate (Sigma Aldrich) were dissolved in Neurobasal medium (Thermo Fisher Scientific) and added before hypoxia at different concentrations (5–10–20 mM of l-lactate and 10 mM of d-lactate). Medium pH was evaluated by using a pH test paper Duotest pH 5.0–8.0 (Macherey–Nagel). We found that pH was not altered by lactate addition up to 20 mM (data not shown).

### MEAs recordings and analysis

Recordings of neuronal electrophysiological activity were performed with 24-well MEAs (Multichannel systems) (24 independent wells with 12 embedded microelectrodes, 30 μm in diameter and spaced 200 μm apart) covered with a Breathe Easier sealing membrane (Sigma Aldrich). Data were acquired with the Multiwell Screen software (Multi Channel Systems, Reutlingen, Germany) at a frequency of 10 kHz and signals were filtered between 100 and 3500 Hz. Data analysis was performed with the use of the Multiwell Analyzer software (Multi Channel Systems, Reutlingen, Germany) in combination with custom made MATLAB scripts (The Mathworks, Natick, MA, USA).

#### Spike detection

Spikes exhibited by neurons were detected if exceeding a threshold of 4.5 times the standard deviation of the baseline noise. An electrode was considered active if exhibiting at least 0.1 spikes/s. The mean firing rate (MFR) was calculated as the average of the number of spikes in time per electrode in a well, considering only the active electrodes.

#### Burst detection

Single channel bursts were detected when containing a minimum of 4 spikes with an inter spike interval between 50 and 100 ms. The minimum interval between bursts and burst duration were set at 100 ms and 50 ms, respectively. A channel was defined as bursting channel if exhibiting at least 0.4 burst/min.

#### Network burst detection

A network burst was defined as temporally overlapping single-channel bursts when the following qualifications apply: the number of distinct bursting channels needed to be at least 8 and at some time during the sequence of time at least 8 channels were simultaneously bursting (i.e. burst with temporal overlap > 0 ms with another burst). The network burst rate (NBR) was calculated as the number of network bursts detected in time in a well. The network burst duration (NBD) was calculated as the average duration of all network bursts detected in a well.

For each well, MFR, NBR and NBD were extracted for every timepoint and normalized with respect to the values from the normoxia phase. Afterwards, the average of normalized MFR, NBR and NBD was calculated for every timepoint among the wells with the same experimental condition. In experiments with neuronal and astrocyte activation, the parameters were evaluated only during the time windows in which stimulation was not delivered.

### Live/dead assays

CellEvent™ Caspase-3/7 Green Detection Reagent (Invitrogen) (1:500) was added to each well before the beginning of experiments, and incubated for 30 min at 37 °C for detection of apoptotic cells (green). In neuronal networks not expressing ChR2, after the end of experiments Propidium Iodide (PI) (Invitrogen) (1:1000) was added to each well and incubated for 15 min at room temperature (RT) to detect dead cells (red). Then, coverslips were washed with PBS and fixated with 3.7% paraformaldehyde (PFA) for 15 min at RT. After washing with PBS, DAPI (Sigma Aldrich) (1:1000) was added into each well and incubated for 20 min at RT to stain cellular nuclei (blue). In neuronal networks expressing ChR2, apoptotic and dead cells were detected separately. Apoptotic cells were detected with CellEvent™ Caspase-3/7 Green Detection Reagent (Invitrogen) (1:500), as previously described. Dead cells were detected with a ReadyProbes™ Cell Viability Imaging Kit Blue/Green (Invitrogen): after the end of experiments, two drops per ml of NucBlue™ Live and NucGreen™ Dead reagents were added into each well and incubated for 15/30 min at RT to stain the nuclei of all cells (blue) and detect dead cells (green), respectively. Then, coverslips were washed with PBS and fixated with 3.7% paraformaldehyde (PFA) for 15 min at RT.

Coverslips were finally washed one more time with PBS and mounted with Mowiol on glass microscope slides. Coverslips were kept in the dark at RT for one night, then they were stored at 4 °C. Epifluorescent pictures (n = 8 per coverslip) were taken at a 40 × magnification (0.085 µm/pixel) with the use of a Nikon Eclipse 50i epifluorescence microscope (Nikon, Japan) and NIS-Elements Microscope Imaging Software (Nikon).

A custom made MATLAB script (The Mathworks, Natick, MA, USA) was used to set a fluorescence intensity threshold for each staining. Then, the total number of cells (DAPI or NucBlue™ Live positive, blue), the number of apoptotic cells (CellEvent™ Caspase-3/7 positive, green) and dead cells (PI positive, red, or both CellEvent™ Caspase-3/7 and PI positive, yellow, or NucGreen™ positive, green) were counted. The number of live cells was calculated by subtracting the apoptotic and dead cells to the total number of cells. The number of live, apoptotic and dead cells was considered as percentage of the total number of cells.

### Immunostaining

Neuronal networks were fixed with 3.7% paraformaldehyde (Sigma Aldrich) for 15 min at RT, washed with PBS (Sigma Aldrich) and stored at 4 °C in PBS until stained. Samples were permeabilized with 0.2% Triton X-100 (Sigma Aldrich) in PBS for 5 min at RT, washed with PBS and blocked with 2% BSA (Sigma Aldrich) in PBS for 30 min at RT to block non-specific binding sites cells. The cultures were stained for rabbit anti-MAP2 (1:1000; Sigma M3696), rabbit anti-GFAP (1:500; Abcam ab7260) overnight at 4 °C in blocking buffer. Samples were washed with PBS and stained with secondary antibodies for 1 h at RT, washed again and as a last step the nuclei were stained with DAPI (1:1000; Sigma Aldrich) 20 min at RT. Samples were washed and mounted with Mowiol (Sigma Aldrich). The secondary antibodies used were goat anti-mouse Alexa Fluor 488 (1:2000, Invitrogen A-11029) and goat anti-rabbit Alexa Fluor 568 (1:2000, Invitrogen A-11036). Images were taken at a 40 × magnification with the use of a Nikon Eclipse 50i Epi-Fluorescence microscope (Nikon, Japan).

### Colorimetric assays

To measure lactate concentration in the culture medium, a Lactate Colorimetric Assay Kit (Abcam, ab65333) was used according to protocol of the manufacturer. Samples of medium were centrifuged, and supernatant was subsequently filtered over a 10 kD cut-off concentrator (Pierce, 88513) to remove proteins and debris. Results were analysed at specific light wavelengths in a Multiskan 60 (Thermo Scientific) plate reader. To make sure all read values were in the range of the calibration curve, series dilutions were made from the samples.

### Statistical analysis

Statistical analysis was performed with the use of GraphPad 9 (GraphPad Software, Inc., CA, USA). Normal distribution of data was ensured using the Kolmogorov–Smirnov normality test. Statistical analysis was performed with the parametric *t*-test or the non-parametric Mann Whitney test (for comparisons between two groups) and the two-way ANOVA test with post-hoc Bonferroni correction (for comparisons between more than two groups). To evaluate statistical significance p-values < 0.05 were considered to be significant. Exact p-values are reported in Supplementary Tables S1–S4. Data are presented as mean ± standard error of the mean (SEM). The number of independent neuronal networks included for each experiment is reported in the figure legends. Graphs were created with the use of OriginLab 2019b (OriginLab Corporation).

### Supplementary Information

Supplementary Information.

## Data Availability

The datasets generated during and/or analysed during the current study are available from the corresponding author on reasonable request.
