# Supplementary material for: Neuroprotective role of lactate in a human in vitro model of the ischemic penumbra
Source: Sci Rep. 2024 Apr 4;14:7973. doi: 10.1038/s41598-024-58669-5 (PMC10994928; doi:10.1038/s41598-024-58669-5)
Supplement: Supplementary file 1 — Supplementary Information. [file 41598_2024_58669_MOESM1_ESM.docx]

**Neuroprotective role of lactate in a human *in vitro* model of the ischemic penumbra.**

Marta Cerina^1,2^, Marloes Levers^1^, Jason M. Keller^3^, Monica Frega^1,4*^

^1^Department of Clinical Neurophysiology, University of Twente, 7522 NB Enschede, the Netherlands

^2^Department of Biotechnology and Biosciences, University of Milano-Bicocca, piazza della Scienza 2, Milano 20126, Italy

^3^BioNTech SE, An d. Goldgrube 12, 55131 Mainz, Germany

^4^Department of Human Genetics, Radboudumc, Donders Institute for Brain, Cognition, and Behaviour, 6500 HB Nijmegen, the Netherlands

*Corresponding Author

dr. Monica Frega

Email: [m.frega@utwente.nl](mailto:m.frega@utwente.nl)

**Supplementary information**


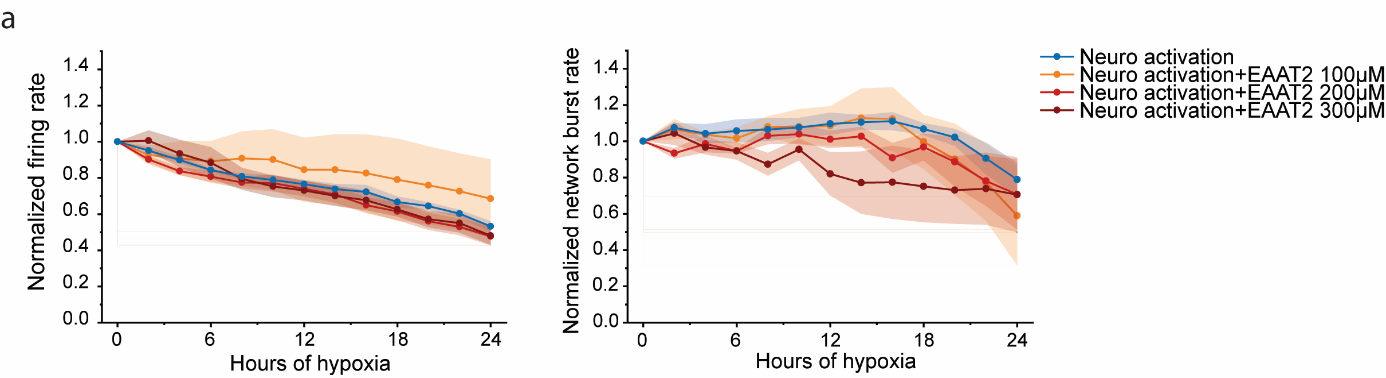


**Supplementary Figure 1.** Graphs showing the effect of EAAT2 blocker on MFR and NBR in neuronal networks activated with optogenetic stimulation. The values are normalized to the data of normoxia (neuro activation n=5, neuro activation + EAAT2 blocker 100 μM n=4, neuro activation + EAAT2 blocker 200 μM n=4, neuro activation + EAAT2 300 μM n=5). Two-way ANOVA test with multiple comparisons and post hoc Bonferroni correction was performed between conditions.


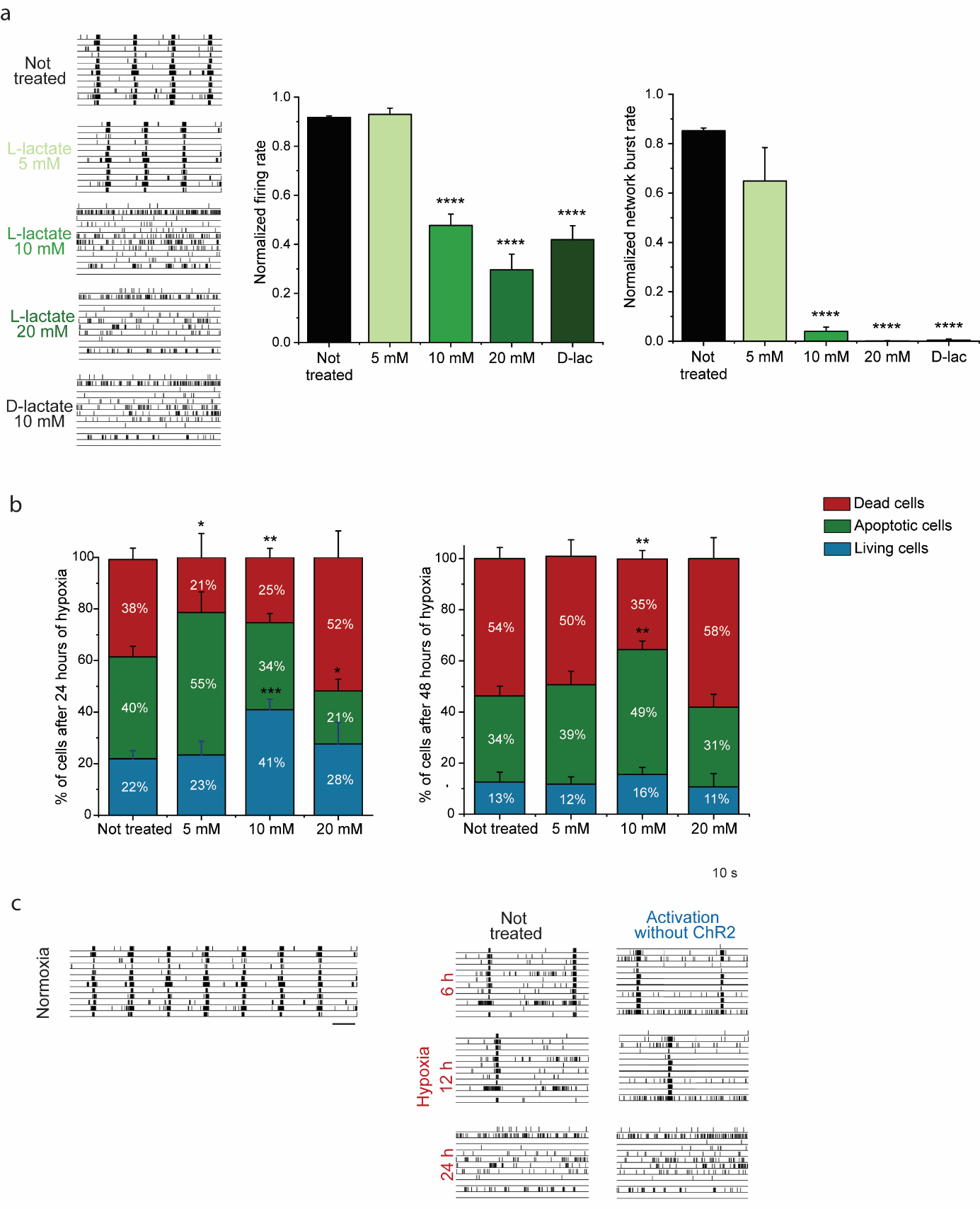


**Supplementary Figure 2. a** Representative raster plots showing 60 seconds of electrophysiological activity exhibited in normoxia by neuronal networks treated with different concentrations of lactate, and bar graphs showing the effect of lactate addition on MFR and NBR in normoxia. The values are normalized to the data of the baseline phase before lactate addition (not treated n=7, L-lactate 2 mM n=5, L-lactate 5 mM n=5, L-lactate 10 mM n=8, L-lactate 20 mM n=5, D-lactate 10 mM n=2). ***p<0.0001, two-way ANOVA test and post hoc Bonferroni correction was performed between conditions. **b** Stacked column graphs showing the percentage of live, apoptotic and dead cells after 24 and 48 hours of hypoxia (not treated n=56 pictures per 7 cultures, L-lactate 5 mM n=16 pictures per 2 cultures, L-lactate 10 mM n=40 pictures per 5 cultures, L-lactate 20 mM n=16 pictures per 2 cultures). *p<0.05, **p< 0.005, ***p<0.0005, unpaired t test or Mann Whitney test was performed between conditions.


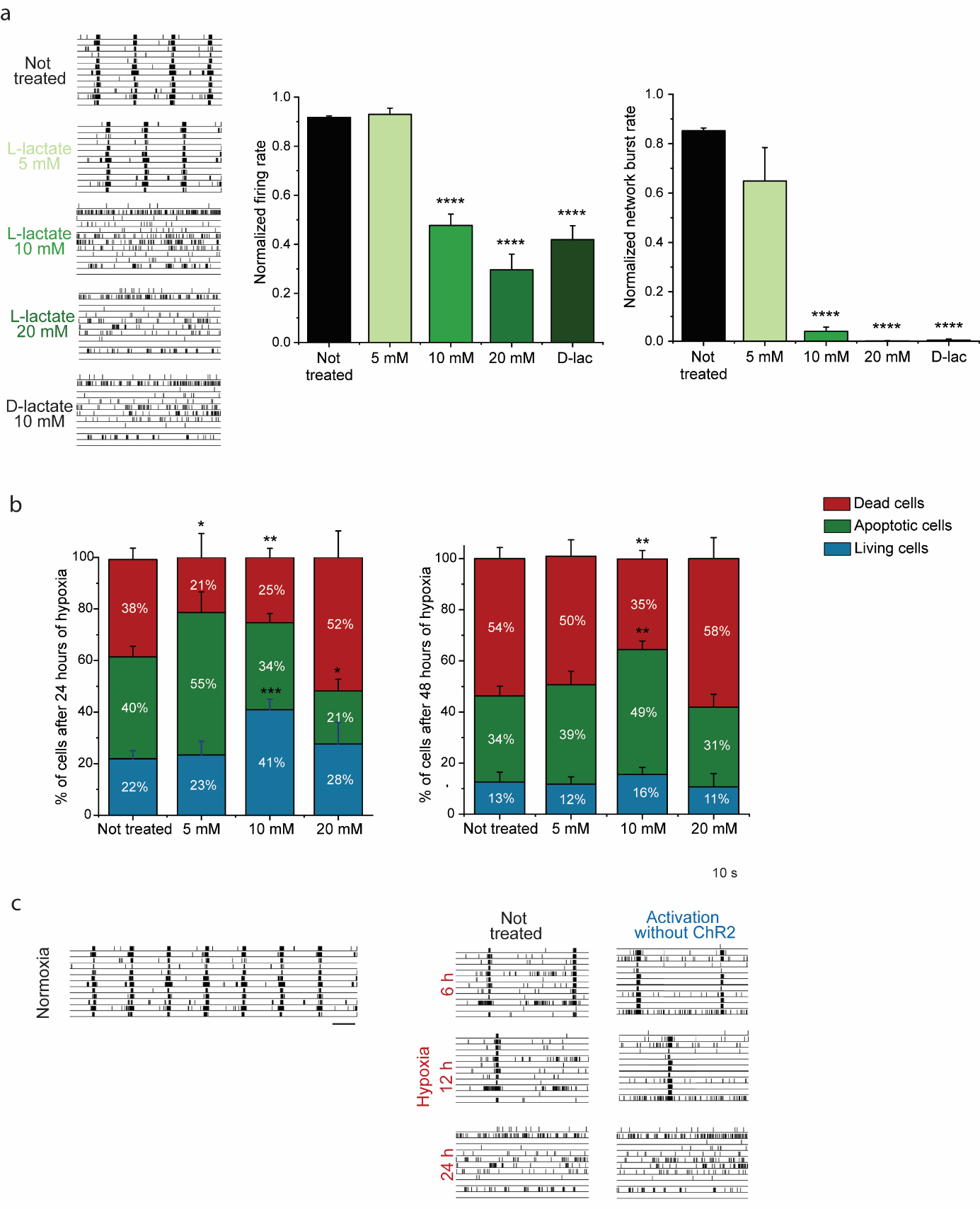


**Supplementary Figure 3.** Representative raster plots showing 60 seconds of electrophysiological activity exhibited at different timepoints of hypoxia (6, 12, 24 hours) by neuronal networks not treated and activated with optogenetic stimulation in absence of ChR2 transduction (neuro activation).

| **Figure** | **Panel** | **Parameter and comparison** | | **Hours of hypoxia** |  | **p-value** |
| --- | --- | --- | --- | --- | --- | --- |
| ***1*** | ***e*** | ***Firing rate***  ***Not treated vs. neuro activation*** | 0 | | ns | >0.9999 |
|  |  |  | 2 | | ns | 0.2642 |
|  |  |  | 4 | | ns | 0.4268 |
|  |  |  | 6 | | ns | 0.5173 |
|  |  |  | 8 | | ns | 0.6832 |
|  |  |  | 10 | | ns | 0.7915 |
|  |  |  | 12 | | ns | 0.5464 |
|  |  |  | 14 | | ns | 0.3790 |
|  |  |  | 16 | | ns | 0.2155 |
|  |  |  | 18 | | ns | 0.4034 |
|  |  |  | 20 | | ns | 0.3619 |
|  |  |  | 22 | | ns | 0.3882 |
|  |  |  | 24 | | ns | >0.9999 |
|  |  | ***Network burst rate***  ***Not treated vs. neuro activation*** | 0 | | ns | >0.9999 |
|  |  |  | 2 | | **** | <0.0001 |
|  |  |  | 4 | | **** | <0.0001 |
|  |  |  | 6 | | **** | <0.0001 |
|  |  |  | 8 | | **** | <0.0001 |
|  |  |  | 10 | | **** | <0.0001 |
|  |  |  | 12 | | **** | <0.0001 |
|  |  |  | 14 | | **** | <0.0001 |
|  |  |  | 16 | | **** | <0.0001 |
|  |  |  | 18 | | **** | <0.0001 |
|  |  |  | 20 | | **** | <0.0001 |
|  |  |  | 22 | | **** | <0.0001 |
|  |  |  | 24 | | **** | <0.0001 |
|  | ***g*** | ***Apoptotic cells***  ***Not treated vs. neuro activation*** | 24 | | * | 0.0237 |
|  |  | ***Dead cells***  ***Not treated vs. neuro activation*** | 24 | | ns | 0.0571 |
|  | ***j*** | ***Firing rate***  ***Neuro activation vs. neuro activation + EAAT_2_ blocker*** | 0 | | ns | >0.9999 |
|  |  |  | 2 | | ns | >0.9999 |
|  |  |  | 4 | | ns | >0.9999 |
|  |  |  | 6 | | ns | >0.9999 |
|  |  |  | 8 | | ns | >0.9999 |
|  |  |  | 10 | | ns | >0.9999 |
|  |  |  | 12 | | ns | >0.9999 |
|  |  |  | 14 | | ns | >0.9999 |
|  |  |  | 16 | | ns | >0.9999 |
|  |  |  | 18 | | ns | >0.9999 |
|  |  |  | 20 | | ns | >0.9999 |
|  |  |  | 22 | | ns | >0.9999 |
|  |  |  | 24 | | ns | >0.9999 |
|  |  | ***Firing rate***  ***Neuro activation vs. neuro activation + MCT_s_ blocker*** | 0 | | ns | >0.9999 |
|  |  |  | 2 | | ns | >0.9999 |
|  |  |  | 4 | | ns | >0.9999 |
|  |  |  | 6 | | ns | >0.9999 |
|  |  |  | 8 | | ns | >0.9999 |
|  |  |  | 10 | | ns | >0.9999 |
|  |  |  | 12 | | ns | >0.9999 |
|  |  |  | 14 | | ns | >0.9999 |
|  |  |  | 16 | | ns | >0.9999 |
|  |  |  | 18 | | ns | >0.9999 |
|  |  |  | 20 | | ns | >0.9999 |
|  |  |  | 22 | | ns | >0.9999 |
|  |  |  | 24 | | ns | >0.9999 |
|  |  | ***Network burst rate***  ***Neuro activation vs. neuro activation + EAAT_2_ blocker*** | 0 | | ns | >0.9999 |
|  |  |  | 2 | | ns | >0.9999 |
|  |  |  | 4 | | ns | >0.9999 |
|  |  |  | 6 | | ns | >0.9999 |
|  |  |  | 8 | | ns | >0.9999 |
|  |  |  | 10 | | ns | >0.9999 |
|  |  |  | 12 | | ns | 0.9182 |
|  |  |  | 14 | | ns | 0.3882 |
|  |  |  | 16 | | ns | 0.3670 |
|  |  |  | 18 | | ns | 0.5107 |
|  |  |  | 20 | | ns | 0.7371 |
|  |  |  | 22 | | ns | >0.9999 |
|  |  |  | 24 | | ns | >0.9999 |
|  |  | ***Network burst rate***  ***Neuro activation vs. neuro activation + MCT_s_ blocker*** | 0 | | ns | >0.9999 |
|  |  |  | 2 | | ns | >0.9999 |
|  |  |  | 4 | | ns | >0.9999 |
|  |  |  | 6 | | ns | 0.3683 |
|  |  |  | 8 | | ns | >0.9999 |
|  |  |  | 10 | | ns | 0.5341 |
|  |  |  | 12 | | ns | 0.1161 |
|  |  |  | 14 | | * | 0.0444 |
|  |  |  | 16 | | ns | 0.0698 |
|  |  |  | 18 | | * | 0.0306 |
|  |  |  | 20 | | * | 0.0211 |
|  |  |  | 22 | | ** | 0.0088 |
|  |  |  | 24 | | ** | 0.0080 |

**Table S1**. Statistical analysis relative to Figure 1.

| **Figure** | **Panel** | **Parameter and comparison** | **Hours of hypoxia** |  | **p-value** |
| --- | --- | --- | --- | --- | --- |
| ***2*** | ***c*** | ***Living cells***  ***Not treated vs. L-lactate*** | 24 | *** | 0.0002 |
|  |  |  | 48 | ns | 0.0741 |
|  |  | ***Apoptotic cells***  ***Not treated vs. L-lactate*** | 24 | ns | 0.4464 |
|  |  |  | 48 | ** | 0.0033 |
|  |  | ***Dead cells***  ***Not treated vs. L-lactate*** | 24 | ** | 0.0047 |
|  |  |  | 48 | ** | 0.0010 |
|  |  | ***Living cells***  ***Not treated vs. D-lactate*** | 24 | ns | 0.1301 |
|  |  |  | 48 | * | 0.0296 |
|  |  | ***Apoptotic cells***  ***Not treated vs. D-lactate*** | 24 | * | 0.0196 |
|  |  |  | 48 | ns | 0.0532 |
|  |  | ***Dead cells***  ***Not treated vs. D-lactate*** | 24 | ns | 0.1047 |
|  |  |  | 48 | ns | 0.8959 |
|  | ***f*** | ***Lactate concentration***  ***Not treated vs. Astro activation*** | 0 | ** | 0.0017 |
|  | ***h*** | ***Firing rate***  ***Not treated vs. astro activation*** | 0 | ns | >0.9999 |
|  |  |  | 2 | **** | <0.0001 |
|  |  |  | 4 | **** | <0.0001 |
|  |  |  | 6 | **** | <0.0001 |
|  |  |  | 8 | **** | <0.0001 |
|  |  |  | 10 | **** | <0.0001 |
|  |  |  | 12 | **** | <0.0001 |
|  |  |  | 14 | **** | <0.0001 |
|  |  |  | 16 | **** | <0.0001 |
|  |  |  | 18 | **** | <0.0001 |
|  |  |  | 20 | **** | <0.0001 |
|  |  |  | 22 | **** | <0.0001 |
|  |  |  | 24 | *** | 0.0002 |
|  | ***i*** | ***Network burst rate***  ***Not treated vs. astro activation*** | 0 | ns | >0.9999 |
|  |  |  | 2 | **** | <0.0001 |
|  |  |  | 4 | **** | <0.0001 |
|  |  |  | 6 | **** | <0.0001 |
|  |  |  | 8 | **** | <0.0001 |
|  |  |  | 10 | **** | <0.0001 |
|  |  |  | 12 | **** | <0.0001 |
|  |  |  | 14 | **** | <0.0001 |
|  |  |  | 16 | **** | <0.0001 |
|  |  |  | 18 | **** | <0.0001 |
|  |  |  | 20 | **** | <0.0001 |
|  |  |  | 22 | **** | <0.0001 |
|  |  |  | 24 | **** | <0.0001 |
|  | ***j*** | ***Network burst duration***  ***Not treated vs. astro activation*** | 0 | ns | >0.9999 |
|  |  |  | 2 | ns | 0.1329 |
|  |  |  | 4 | * | 0.0179 |
|  |  |  | 6 | ** | 0.0028 |
|  |  |  | 8 | ** | 0.0048 |
|  |  |  | 10 | ** | 0.0027 |
|  |  |  | 12 | *** | 0.0006 |
|  |  |  | 14 | *** | 0.0001 |
|  |  |  | 16 | *** | 0.0001 |
|  |  |  | 18 | *** | 0.0007 |
|  |  |  | 20 | *** | 0.0007 |
|  |  |  | 22 | * | 0.0217 |
|  |  |  | 24 | ns | 0.0766 |
|  | ***g*** | ***Apoptotic cells***  ***Not treated vs. neuro activation*** | 24 | ** | 0.0031 |
|  |  | ***Dead cells***  ***Not treated vs. neuro activation*** | 24 | ns | 0.8383 |

**Table S2**. Statistical analysis relative to Figure 2.

| **Figure** | **Panel** | **Parameter and comparison** | | **Hours of hypoxia** |  | **p-value** |
| --- | --- | --- | --- | --- | --- | --- |
| ***S1*** |  | ***Firing rate***  ***Neuro activation vs. neuro activation + EAAT_2_ blocker 100µM*** | 0 | | ns | >0.9999 |
|  |  |  | 2 | | ns | >0.9999 |
|  |  |  | 4 | | ns | >0.9999 |
|  |  |  | 6 | | ns | >0.9999 |
|  |  |  | 8 | | ns | >0.9999 |
|  |  |  | 10 | | ns | >0.9999 |
|  |  |  | 12 | | ns | >0.9999 |
|  |  |  | 14 | | ns | >0.9999 |
|  |  |  | 16 | | ns | >0.9999 |
|  |  |  | 18 | | ns | >0.9999 |
|  |  |  | 20 | | ns | >0.9999 |
|  |  |  | 22 | | ns | >0.9999 |
|  |  |  | 24 | | ns | >0.9999 |
|  |  | ***Firing rate***  ***Neuro activation vs. neuro activation + EAAT_2_ blocker 200µM*** | 0 | | ns | >0.9999 |
|  |  |  | 2 | | ns | >0.9999 |
|  |  |  | 4 | | ns | >0.9999 |
|  |  |  | 6 | | ns | >0.9999 |
|  |  |  | 8 | | ns | >0.9999 |
|  |  |  | 10 | | ns | >0.9999 |
|  |  |  | 12 | | ns | >0.9999 |
|  |  |  | 14 | | ns | >0.9999 |
|  |  |  | 16 | | ns | >0.9999 |
|  |  |  | 18 | | ns | >0.9999 |
|  |  |  | 20 | | ns | >0.9999 |
|  |  |  | 22 | | ns | >0.9999 |
|  |  |  | 24 | | ns | >0.9999 |
|  |  | ***Firing rate***  ***Neuro activation vs. neuro activation + EAAT_2_ blocker 300µM*** | 0 | | ns | >0.9999 |
|  |  |  | 2 | | ns | >0.9999 |
|  |  |  | 4 | | ns | >0.9999 |
|  |  |  | 6 | | ns | >0.9999 |
|  |  |  | 8 | | ns | >0.9999 |
|  |  |  | 10 | | ns | >0.9999 |
|  |  |  | 12 | | ns | >0.9999 |
|  |  |  | 14 | | ns | >0.9999 |
|  |  |  | 16 | | ns | >0.9999 |
|  |  |  | 18 | | ns | >0.9999 |
|  |  |  | 20 | | ns | >0.9999 |
|  |  |  | 22 | | ns | >0.9999 |
|  |  |  | 24 | | ns | >0.9999 |
|  |  | ***Network burst rate***  ***Neuro activation vs. neuro activation + EAAT_2_ blocker 100µM*** | 0 | | ns | >0.9999 |
|  |  |  | 2 | | ns | >0.9999 |
|  |  |  | 4 | | ns | >0.9999 |
|  |  |  | 6 | | ns | >0.9999 |
|  |  |  | 8 | | ns | >0.9999 |
|  |  |  | 10 | | ns | >0.9999 |
|  |  |  | 12 | | ns | >0.9999 |
|  |  |  | 14 | | ns | >0.9999 |
|  |  |  | 16 | | ns | >0.9999 |
|  |  |  | 18 | | ns | >0.9999 |
|  |  |  | 20 | | ns | >0.9999 |
|  |  |  | 22 | | ns | >0.9999 |
|  |  |  | 24 | | ns | >0.9999 |
|  |  | ***Network burst rate***  ***Neuro activation vs. neuro activation + EAAT_2_ blocker 200µM*** | 0 | | ns | >0.9999 |
|  |  |  | 2 | | ns | >0.9999 |
|  |  |  | 4 | | ns | >0.9999 |
|  |  |  | 6 | | ns | >0.9999 |
|  |  |  | 8 | | ns | >0.9999 |
|  |  |  | 10 | | ns | >0.9999 |
|  |  |  | 12 | | ns | >0.9999 |
|  |  |  | 14 | | ns | >0.9999 |
|  |  |  | 16 | | ns | 0.6079 |
|  |  |  | 18 | | ns | >0.9999 |
|  |  |  | 20 | | ns | >0.9999 |
|  |  |  | 22 | | ns | >0.9999 |
|  |  |  | 24 | | ns | >0.9999 |
|  |  | ***Network burst rate***  ***Neuro activation vs. neuro activation + EAAT_2_ blocker 300µM*** | 0 | | ns | >0.9999 |
|  |  |  | 2 | | ns | >0.9999 |
|  |  |  | 4 | | ns | >0.9999 |
|  |  |  | 6 | | ns | >0.9999 |
|  |  |  | 8 | | ns | >0.9999 |
|  |  |  | 10 | | ns | >0.9999 |
|  |  |  | 12 | | ns | 0.9182 |
|  |  |  | 14 | | ns | 0.3882 |
|  |  |  | 16 | | ns | 0.3670 |
|  |  |  | 18 | | ns | 0.5107 |
|  |  |  | 20 | | ns | 0.7371 |
|  |  |  | 22 | | ns | >0.9999 |
|  |  |  | 24 | | ns | >0.9999 |

**Table S3**. Statistical analysis relative to Supplementary Figure 1.

| **Figure** | **Panel** | **Parameter and comparison** | **Hours of hypoxia** |  | **p-value** |
| --- | --- | --- | --- | --- | --- |
| ***S2*** | ***b*** | ***Firing rate***  ***Not treated vs. 5 mM*** | 0 | ns | >0.9999 |
|  |  | ***Firing rate***  ***Not treated vs. 10 mM*** | 0 | **** | <0.0001 |
|  |  | ***Firing rate***  ***Not treated vs. 20 mM*** | 0 | **** | <0.0001 |
|  |  | ***Firing rate***  ***Not treated vs. D-lac*** | 0 | **** | <0.0001 |
|  |  | ***Network burst rate***  ***Not treated vs. 5 mM*** | 0 | ns | 0.2919 |
|  |  | ***Network burst rate***  ***Not treated vs. 10 mM*** | 0 | **** | <0.0001 |
|  |  | ***Network burst rate***  ***Not treated vs. 20 mM*** | 0 | **** | <0.0001 |
|  |  | ***Network burst rate***  ***Not treated vs. D-lac*** | 0 | **** | <0.0001 |
|  | ***c*** | ***Living cells***  ***Not treated vs. 5 mM*** | 24 | ns | 0.7874 |
|  |  |  | 48 | ns | 0.0817 |
|  |  | ***Apoptotic cells***  ***Not treated vs. 5 mM*** | 24 | ns | 0.0516 |
|  |  |  | 48 | ns | 0.5184 |
|  |  | ***Dead cells***  ***Not treated vs. 5 mM*** | 24 | * | 0.0188 |
|  |  |  | 48 | ns | 0.7090 |
|  |  | ***Living cells***  ***Not treated vs. 10 mM*** | 24 | *** | 0.0002 |
|  |  |  | 48 | ns | 0.0741 |
|  |  | ***Apoptotic cells***  ***Not treated vs. 10 mM*** | 24 | ns | 0.4464 |
|  |  |  | 48 | ** | 0.0033 |
|  |  | ***Dead cells***  ***Not treated vs. 10 mM*** | 24 | ** | 0.0047 |
|  |  |  | 48 | ** | 0.0010 |
|  |  | ***Living cells***  ***Not treated vs. 20 mM*** | 24 | ns | 0.7303 |
|  |  |  | 48 | ns | 0.2700 |
|  |  | ***Apoptotic cells***  ***Not treated vs. 20 mM*** | 24 | * | 0.0497 |
|  |  |  | 48 | ns | 0.7557 |
|  |  | ***Dead cells***  ***Not treated vs. 20 mM*** | 24 | ns | 0.1885 |
|  |  |  | 48 | ns | 0.6470 |

**Table S4**. Statistical analysis relative to Supplementary Figure 2.
